# The role of follicular helper CD4 T cells in the development of HIV-1 specific broadly neutralizing antibody responses

**DOI:** 10.1186/s12977-018-0437-y

**Published:** 2018-08-06

**Authors:** Eirini Moysi, Constantinos Petrovas, Richard A. Koup

**Affiliations:** Immunology Laboratory, Vaccine Research Center, NIAID, NIH, Bethesda, USA

**Keywords:** Germinal center, Tfh, Broadly neutralizing antibody, Vaccines

## Abstract

The induction of HIV-1-specific antibodies that can neutralize a broad number of isolates is a major goal of HIV-1 vaccination strategies. However, to date no candidate HIV-1 vaccine has successfully elicited broadly neutralizing antibodies of sufficient quality and breadth for protection. In this review, we focus on the role of follicular helper CD4 T-cells (Tfh) in the development of such cross-reactive protective antibodies. We discuss germinal center (GC) formation and the dynamics of Tfh and GC B cells during HIV-1/SIV infection and vaccination. Finally, we consider future directions for the study of Tfh and offer perspective on factors that could be modulated to enhance Tfh function in the context of prophylactic vaccination.

## Background

A sterilizing HIV-1 vaccine would greatly facilitate the fight against the HIV-1 epidemic. Research efforts over the past 35 years have afforded unique insights into the biology, virology and immunology of HIV-1 infection including a better appreciation of the importance of cross-clade reactive, broadly neutralizing antibodies (bnAbs) [1, 2]. HIV-1 is a highly diverse pathogen and successfully evades immunity by constantly shifting its antigenicity through evolution [3]. The failure of the Merck adenovirus type 5 (Ad5)-based vaccine in the STEP trial to induce robust protective cell-mediated immunity (CMI) responses to either prevent HIV-1 infection or suppress viral load in infected individuals refocused vaccine development efforts on humoral immunity [4]. bnAbs are antibodies that recognize highly conserved sites of vulnerability in many different circulating strains of HIV-1 [5, 6]. As such, they hold great promise for HIV-1 vaccine development. Studies of passive bnAb transfer in non-human primates and humans have been shown to prevent infection and reduce viral loads, suggesting that combinations of durable bnAb levels could be used prophylactically as well as therapeutically [1, 2, 7–13]. However to date, despite the use of potent immunogens and delivery strategies, efficacy in HIV-1 vaccine trials remains either very low or absent [14–17]. This apparent disconnect between potent immunogen delivery and optimal response elicitation has sparked a renewed interest in the tissue-specific dynamics of bnAb development, including the selection and expansion of specific germline BCR precursors in B cell follicles, and the immunological correlates of those dynamics. Such topics have traditionally been hard to study in lymph node (LN) samples due to the difficulty in obtaining LN material from HIV-1+ individuals. More recently however, the availability of longitudinal biopsies from non-human primates in combination with the advancement of multi-parameter imaging and flow cytometry techniques have opened new avenues for tissue-specific immunity exploration [18, 19]. Here, we review the recent literature on Tfh cells and bnAbs in the context of chronic HIV-1/SIV infection and vaccination and offer perspective on open questions that need to be addressed in order to design vaccine strategies that will optimally engage the humoral arm of the adaptive immune system.

## Tfh cells and their role in GC responses

**Fig. 1:**
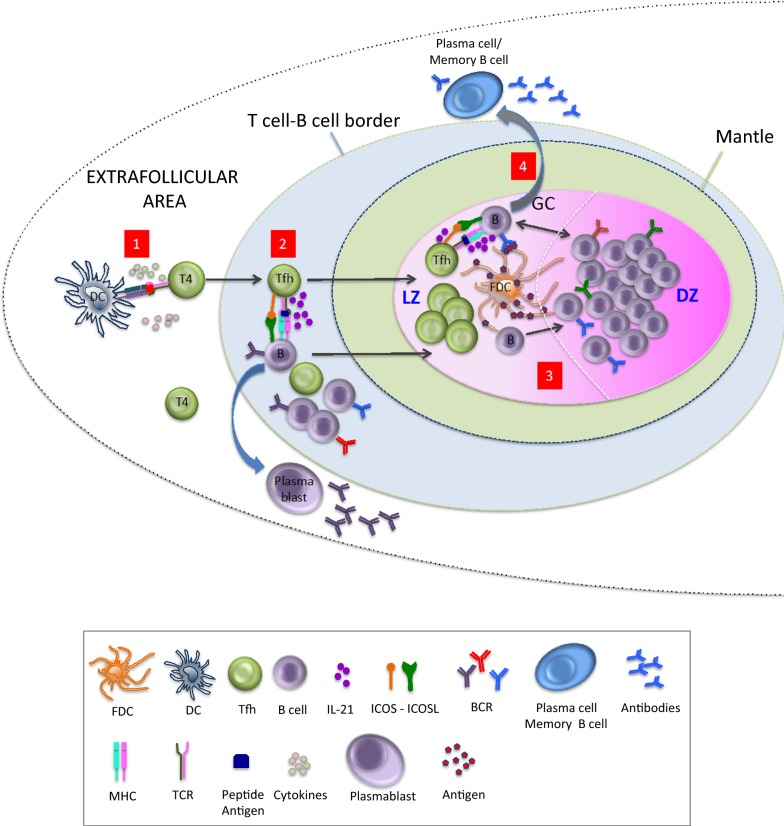
Sequence of events leading to GC induction and the production of high affinity antibodies. (1) The induction of Tfh takes place in the T-cell zone upon interaction with DCs. (2) In the T–B border, B cells present antigen in complex with MHC-II to Tfh. These early cognate interactions determine which B cells will migrate to the GC to undergo somatic hypermutation and clonal expansion and which will become plasmablasts. (3) GC B cells constantly migrate between the LZ and DZ sampling antigen on FDCs and receiving help from Tfh. The nature of these interactions determines which GC B cells will survive and become plasma cells as well as the number of rounds of affinity maturation and somatic hypermutation a B cell will undergo before selection and exit to the periphery. (4) GC B cells become either antibody secreting plasma cells or memory B cells upon GC exit. Tfh, follicular T-helper cell; GC, germinal center; DC, dendritic cell; MHC, major histocompatibility complex; LZ, light zone; DZ, dark zone; FDC, follicular dendritic cell; BCR, B cell receptor; ICOS, Inducible T-cell costimulator; TCR, T cell receptor

Tfh are cells that localize to the lymph nodes, within well-defined structures called B-cell follicles (Fig. 1) [20, 21]. They are critical for the maturation, isotype switching, and somatic hypermutation (SHM) of B cells as well as for the survival of memory B cells and antibody-secreting plasma cells [20, 22, 23]. Their role thus is instrumental for the generation of high affinity antibodies. Tfh cells express low levels of CCR7 and are classically defined by the expression of the surface receptors CXCR5 and costimulatory receptors PD-1 and ICOS [20]. Their unique phenotype is preserved among different species including mice [24], non-human primates [25] and humans [21]. Although their ontogeny is not entirely clear, Tfh cells share characteristics with other CD4 T-cell lineages [26, 27]. However, their transcriptional regulation and gene expression profiles are distinct from all other lineages such as Th1, Th2, Th17 and regulatory T cells [28, 29]. Maturation of Tfh cells begins with antigen priming by DCs in the T cell zones surrounding the lymphoid follicles [30] and continues at the follicular T-B border with cognate interactions between Tfh and B-cells [31, 32]. These events lead to the induction of the transcription factor Bcl-6 as well as c-Maf that control lineage commitment to the Tfh fate [33, 34]. These early Tfh-B cell interactions require expression of the surface receptors ICOS, OX40 and CD40-ligand as well as expression of the cytokines IL-4 and IL-21 and have been shown to influence both Tfh fate commitment and the survival and ability of B cells to enter the GC response [29, 35–37]. B-cells activated during these early Tfh-B cell cognate interactions can subsequently move in extrafollicular areas for proliferation and differentiation into short-lived, antibody-secreting plasma cells or migrate into B cell follicles to establish a GC [38]. What determines either fate is not entirely clear but evidence exists to suggest that the decision might be contingent on the affinity of the B cell receptor (BCR) for the foreign antigen [39, 40], the density of antigen-MHC class II complex engagement [41], and the costimulatory signals received from T cells [38]. In these early steps of GC formation, the relative density of MHC class II expression on B cells appears to reflect the affinity of a given BCR precursor for antigen and the efficiency of BCR-mediated antigen uptake [42]. Thus, early cognate Tfh-B cell interactions may represent an important bottleneck in the ability of Tfh to recruit B cells of a given specificity into the response [43]. The follicular recruitment, frequency and function of Tfh, is additionally influenced by the relative abundance of antigen and availability of chemokines such as CXCL13 and SDF-1 [44]. In the GC, B cells constantly migrate between the light zone (LZ) and the dark zone (DZ) and thus the process of GC selection is highly regulated spatiotemporally [45]. T cell help in the LZ has been shown to activate the mTORC1 pathway, promoting a phase of anabolic growth that precedes and sustains the successive cycles of DZ proliferation [46]. Thus, Tfh in the LZ determine the cycling speed and number of cell divisions that a GC B cell will undergo as well as the associated number of B cell receptor (BCR) mutations in the GC per round of selection [43, 47, 48]. These data suggest that optimal GC reactivity and bnAb development depend on the phenotype of Tfh, as well as their spatiotemporal localization.

## HIV-1/SIV infection and bnAb development

### Role of Tfh cell quality

HIV-1/SIV infection and the resulting viremia influence the signals and mechanisms that regulate the dynamics of Tfh cells as well as the dynamics of Tfh-GC B cell interaction in LN follicles. Tfh induction can be traced as early as 14 days post-infection in NHPs challenged with SIV [49] and studies in humans and NHPs show that despite CD4 T cells being depleted during chronic HIV-1/SIV infection, the frequency of CXCR5+ PD-1^hi^ CD4 T cells significantly increases both in the blood as well as in the LNs [50–53]. However, the increase in the frequencies of Tfh is not directly translated into higher bnAb levels. Only 20–30% of infected individuals are capable of mounting broadly neutralizing antibodies with HIV-1-specificities that have the potential to bind multiple HIV-1 envelope spikes of heterologous lineage during the first three years of infection [54]. Why some individuals and animals are able to develop bnAbs in the context of viremia whereas others do not is not entirely clear but both virologic, genetic and immunologic factors seem to influence this outcome. Virologic parameters that have been linked to bnAb production include characteristics of the infecting strain (ie viral loop length) [55–57] and degree of viral diversity [56]. For instance, exposure to multiple variants, as in the case of superinfection, has been shown to predict the development of bnAbs [56, 58] and studies in NHP and humans point to antigenic diversity (ie Env) being an important parameter with high viral loads and greater sequence evolution predicting a greater breadth of neutralization [56, 58, 59]. Host genetic factors, such as expression of specific HLA alleles have also been associated with bnAb activity in some cohorts [60, 61] whereas from an immunological stand-point, two parameters considered important are the ability of Tfh cells to provide help to B cells [62] and level of T-cell regulation [63].

**Fig. 2:**
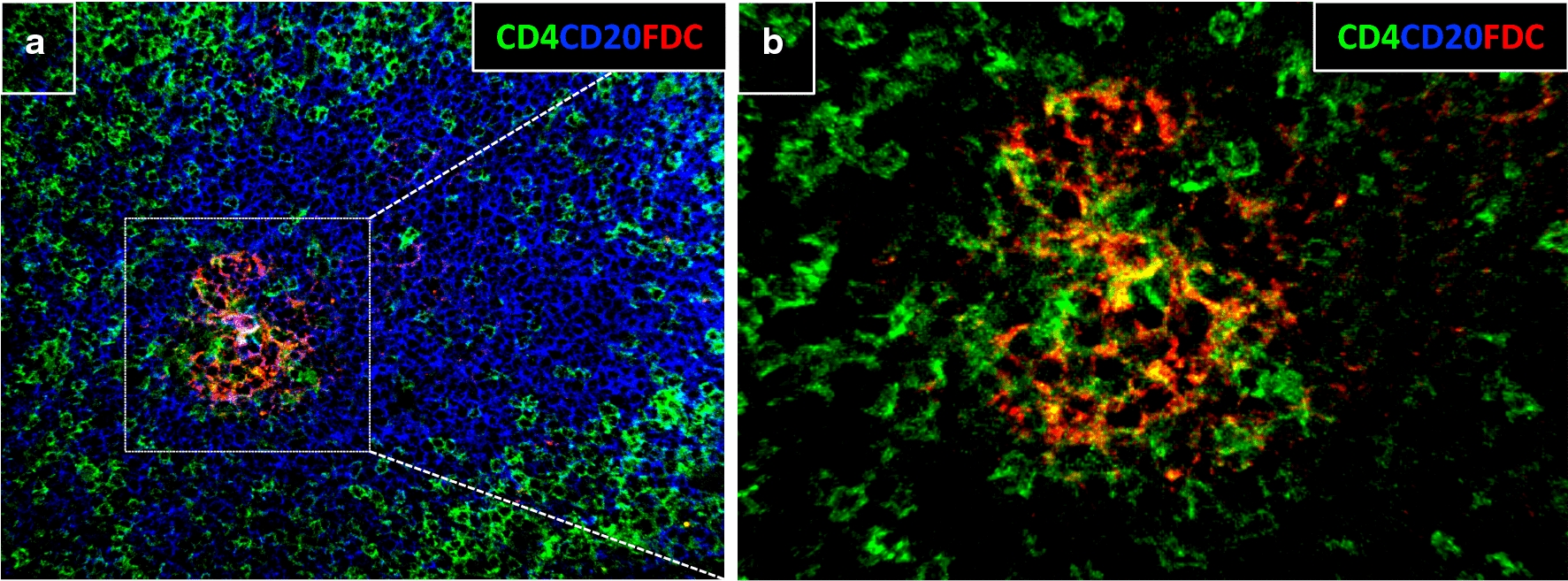
Convergence of CD4+ T cells, B cells and FDC in a B cell follicle. Confocal imaging microscopy showing the convergence of immune populations contributing to the development of bnAbs in a lymph node B cell follicle derived from a HIV- individual. CD4 T cells are shown in green, CD20 in blue and FDCs in red. Images were acquired at ×40 (NA 1.3). Captions are **a** 50 μm and **b**15 μm respectively

CD4 T cells in the LN are a major target for HIV-1 infection. CXCR5+ PD-1^hi^ cells in infected LNs have been shown to harbor a significantly increased frequency of HIV-1 DNA compared with non-Tfh cells [52] and to represent a major reservoir of latent virus in humans receiving antiretroviral therapy [64, 65]. In addition, their localization in close proximity to virion-ladden follicular dendritic cells (FDCs) in B cell follicles makes them increasingly susceptible to infection (Fig. 2) [66, 67]. Tfh cells isolated from HIV-1-infected patients produce less IL-21, a critical cytokine for GC formation, GC B cell proliferation and B cell maturation [68] . Exogenous administration of IL-21 has been shown to improve memory B cell frequencies, which suggests that IL-21 deficiency may, at least in part, impair the formation of memory B cell responses [69, 70]. HIV-1/SIV infection also imparts defects in the PD-1/PD-L1 axis. GC B cells from HIV-1 infected individuals express elevated levels of PD-L1 and have been shown to reduce ICOS and IL-21 expression in Tfh cells upon PD-1 ligation which could further compromise their ability to provide help to B cells [62]. The in vivo cycling capacity of Tfh cells is also compromised compared with other CD4 T-cell populations within the lymph nodes of infected NHP [53]. Moreover, in chronic untreated HIV-1+ infection Tfh become functionally skewed and oligoclonally restricted [71] Thus, HIV-1/SIV infection potentially alters the ability of Tfh to provide help to GC B-cells through a number of mechanisms. However, to what extend tissue-specific Tfh responses, including ICOS, CD40L expression and cytokine secretion differ between broadly neutralizers and non-neutralizers remains poorly understood. More recently, a number of studies have pointed to the heterogeneity of the Tfh population within the GC but less is known about the exact ontogeny of these individual phenotypes [72–74]. For instance, Tfh cells expressing CD57, show a significantly higher frequency of HIV-1 infection compared with extrafollicular CD4 T cells [75, 76] and transcriptional signatures that show differences when compared to CD57- [72]. Moreover, chronic SIV infection has been shown to promote expansion of CXCR3+ expressing, IFN-γ producing GC Tfh cells (Th1-like) which are functionally distinct from CXCR3− Tfh in terms of phenotype and cytokine production [77]. To what extend these alterations affect the development of bnAbs is not currently known. Differences in the antigen-specificity or clonality of Tfh cells may also account for differences in the HIV-1-specific GC B-cell responses [71]. Even though the in vitro quantification of antigen-specific Tfh cells has been challenging [78] data supporting different roles for phenotypically distinct Tfh cells are available. In one study, IL-4 producing Env-specific Tfh but not those producing IFN-γ favored the development of Env-specific IgG+ GC B cells in NHP challenged with SHIV_AD8_ in the chronic phase [59]. Further research is needed to understand how viral infection modulates the ability of Tfh cells to provide help to B cells, their positioning, Tfh subtype transcriptional differences as well as the factors that contribute to Tfh persistence in the face of chronic viremia.

### Role of antigen and immune inflammation

Broadly neutralizing antibodies have been shown to develop after several years of infection in HIV-1+ individuals, with the first cross-neutralizing antibody responses appearing on average at 2.5 years post- infection [79]. Such bnAbs are characterized by a number of unusual features; they possess high-levels of somatic hypermutation reaching, in some cases, frequencies of 32% and 20% in heavy- and kappa- chain V genes respectively [5], extraordinarily long CDR3 antigen-contacting sites [5, 80, 81] and are poly- or autoreactive [82]. Their unique characteristics, potency and breadth arise through a continuous process of B cell adaptation and affinity maturation which may be fueled by a prolonged exposure to antigen [83, 84]. Antigenic persistence and antigen dose both determine the size and duration of the Tfh response and GC reaction [85] and Tfh cell accumulation in the chronic phase of HIV-1 infection is substantially decreased by ART [51–53]. Therefore, prolonged antigen availability within GCs in the context of HIV-1/SIV may be contributing to bnAb development by affecting both Tfh and B cell dynamics.

### Role of GC B cell quality

Another hallmark of HIV-1/SIV infection is B cell dysregulation [86]. Several B cell abnormalities manifest during HIV-1 infection including phenotypic changes, polyclonal B cell activation and hypergammaglobulinaemia, as well as B cell unresponsiveness to T-cell independent and T-cell dependent B cell activation, all of which might affect the ability of HIV-1 infected individuals to develop bnAbs and respond to therapeutic vaccination or prophylactic vaccination against other infectious diseases such as hepatitis B and influenza [86–91]. The accumulation of Tfh cells in chronic HIV-1 [51, 52] and SIV [53] is associated with expansion of GC B cells and plasma cells. Maturation however into memory B cells is reduced [92]. In addition, B cells from patients with HIV-1 have low expression of the CXCL13 receptor CXCR5 compared with healthy controls and secrete large amounts of CXCL13 upon polyclonal stimulation which could, under physiological conditions alter the homing of B-cells [93]. Currently, there is little information on how B cell impairment affects the bnAb response in HIV-1/SIV infection. A better understanding of (1) the antigen-specific LN B cell responses, (2) the molecular profile and of GC B cell maturation process and (3) the spatial organization of GC immune reactions in the context of HIV-1/SIV are warranted in order to successfully design future vaccination strategies.

### Role of follicular regulatory T-cells (Tfr)

FoxP3+ CD4+ Treg cells play an important role in the regulation of B cell responses as in their absence the levels of circulating antibodies increase [94]. T follicular regulatory (Tfr) cells, are a subset of FoxP3+ CD4+ Treg cells that localize to the GC during immune responses to control the magnitude of the response [95]. Phenotypically, Tfr express CXCR5+ alongside the classical Treg marker CD25 [96] but their exact function in the GC, especially in the context of HIV-1 is not yet clear. Given that FoxP3 is expressed in memory non-Treg CD4 T cells too, further phenotypic characterization of LN Tregs is necessary. Under physiological conditions, a skewed presence of Tfr cells in extrafollicular areas compared to follicles has been shown [97]. In chronic HIV-1/SIV infection, the absolute number of Tfr cells within total LN CD4 T cells is increased [98, 99]. However how this may be impacting upon neutralizing B- cell development remains to be found. Studies in LNs of NHP, have shown an inverse correlation of the frequency of LN Tfh cells with Tfr frequency and the avidity of antibodies recognizing the SIV gp120 protein in plasma. Hence, Tfr could act to limit the maturation of antigen-specific responses [100] with bnAb development during HIV-1/SIV infection being favored by a relaxation in the regulatory control of GC antibody production [101, 102]. Further research in NHP LN biopsies and human FNA samples are thus warranted to address in more detail the role of Tfr responses in the expansion of B cells with neutralizing and non-neutralizing reactivities.

## Lessons from vaccination

The realization that many individuals harbor bnAb precursors in their naïve B cell repertoires has reignited the hope that a bnAb-based HIV-1 vaccine might be attainable. Precursor frequency for bnAbs in the naïve repertoire is usually low, with those of the VRC-01 class estimated at ~1 out of 400,000 naïve B cells [103]. In addition, the affinity of such germline precursors for antigen is also low [104]. Thus, one critical question is how to optimally engage these precursors at tissue-level. The introduction of germline-targeting immunogens, namely immunogens aiming at activating B cells that express specific germline BCRs, represents one strategy to tackle low precursor frequencies [105, 106]. Furthermore, immunization studies indicate that for optimal vaccine efficacy the following conditions must be met: (1) B cell precursors must be present in the repertoire at sufficient frequencies [106, 107] (2) B cell precursors must have sufficient affinity for antigen for recruitment into the GC and competitive success [106, 107] (3) B cells and memory B cells must express a favorable antibody class [108] (4) the right structural context and T-B cell stoichiometry must occur in GC for optimal engagement and somatic hypermutation [107] (Table 1).

**Table 1:** Parameters linked to the development of broadly neutralizing antibodies

Parameter	References
Tfh	
Frequency	[50]
Quality	[50, 52, 61, 69]
Phenotype / specificity	[50, 61, 69]
B-cells	
Precursor frequency	[40, 94, 95, 97, 106, 107, 116]
BCR affinity for antigen	[37, 40, 107]
Isotype class	[98]
Amount of help received by Tfh	[36, 40, 44, 52]
Antigen	
Persistence	[76, 106]
Diversity	[69]
Tregs/Tfr	
Frequency	[53, 90, 91]

Tfh cells are central to GC formation and therefore their quantity and quality play a major role. In the absence of T cells, GCs formed in response to T-independent antigens collapse shortly after compartmentalization into the DZ and LZ [38]. To date, most of the data investigating Tfh quality and phenotype in the context of prophylactic vaccination come from circulating Tfh cells (pTfhs). Although the latter are often used as biomarkers of GC activity the lineage relationship between bona fide Tfh in LN and circulating Tfh is not clear [109–111]. The high heterogeneity of pTfh cell phenotypes and gene expression profiles further complicates the interpretation of relevant studies [74, 112, 113]. Of all subsets, PD-1+ CXCR3− CXCR5+ CD4 T cells found in the blood have been found to be the population most related to GC Tfh cells by gene expression, cytokine expression profile and ability to provide help to B cells in vitro [110]. Higher expression of Tfh-associated genes, including CD40L, IL-21 and ICOS has been observed in animals mounting strong neutralizing antibody responses [43] and in the RV144 trial that produced some efficacy in humans, HIV-1-specific IL-21 producing pTfh cells were elevated [102, 110, 114, 115]. In addition, HIV-1 infected individuals with strong neutralizing responses harbor higher frequencies of pTfh [102, 110]. However, an association between pTfh and bnAb development is not always present [109]. Further research is needed to delineate the relationship between GC Tfh, pTfh and bnAbs in the context of prophylactic and therapeutic vaccination.

Antigen presentation and recognition are central to Tfh cell induction [30, 116] Therefore, increasing antigen availability has emerged as a rational approach to enhance Tfh responses for neutralizing antibody production in the context of vaccination [117]. Different strategies are under investigation targeting an effective delivery of immunogens, including (a) the continuous immunogen infusion whereby soluble native antigen degradation is reduced [118, 119], (b) the formation of immuno-complexes and deposition of antigen on monocytes, DCs or FDCs [120, 121], (c) the use of delivery platforms such as nanoparticles, liposomes, viral particles and use of adjuvants that can prolong antigen retention [122]. In parallel, approaches to induce affinity maturation of bnAb-class specific naïve B-cell precursors (ie VRC01 or PGT121-class naïve B-cells) by delivering structurally optimized immunogens in sequential immunization protocols are also being tested [104, 123–125]. Combining such protocols with Tfh-boosting strategies will most likely be necessary for optimal vaccine efficacy. The type of prime-boost strategy also affects ensuing Tfh responses. Prime-boost strategies employing pure DNA instead of protein at priming, have been shown to increase Tfh differentiation, GC reactivity and antigen-specific antibody titers in mice [126] although to what extend they increase specifically broadly neutralizing antibodies remains to be determined. The interval between priming and boosting is also important for optimal Tfh and B-cell kinetics as an early boost, at the time when Tfh and B-cell maturation are still ongoing, could lead to suboptimal responses [127].

Understanding recall responses is also critical. GC B cell sequencing data indicate that memory B cells actively re-circulate after each immunization and reseed new GCs, with moderately mutated memory B cell lineages being more likely to participate in this reseeding. [128]. In a study by Havenar- Daughton et al, GC B cell frequencies in the draining LN in response to the final immunization were found to be the most predictive factor for the development of autologous nAbs with the top neutralizers having three fold more responding GC B cells than animals that only made non-neutralizing Ab responses [128]. Thus, understanding the recall kinetics of Tfh and B-cells in the context of serial immunizations will be key to developing prophylactic and therapeutic HIV-1 vaccines.

## Conclusion

Much progress has been made over recent years in understanding Tfh cells and their implication in GC B cell responses. It is now clear that Tfh cells are instrumental for the generation of high affinity antibodies. Hence, manipulation of this subset and its microenvironment will be necessary for optimal vaccine efficacy. Tfh cell induction and optimal antigen-specific Tfh- B cell interaction will most likely necessitate a combination of more than one strategy. Deeper insights into the dynamics of Tfh cell induction, function and memory are also warranted. To this end, longitudinal studies in individuals with and without neutralizing activity with fine needle aspirates (FNA) could surpass the current limitations of LN biopsies and the need for complete removal of a LN at the site of induction. Powerful system immunology approaches, including bioinformatics and next-generation sequencing to uncover innate signatures and immune mechanisms that correlate with protection and that can improve vaccine induced long-lived neutralizing antibody responses will also be needed to guide the rational development of HIV-1 vaccines. A better understanding of those tissue-specific correlates that lead to robust GC B cell expansion, SHM and neutralization breadth will be key to achieving the goal of sterilizing HIV-1 immunity.
